# Optimizing Postoperative Recovery in Colorectal Surgery: A Systematic Review on the Efficacy of Enhanced Recovery After Surgery (ERAS) Protocols

**DOI:** 10.7759/cureus.90087

**Published:** 2025-08-14

**Authors:** Mohey Aldien Ahmed Elamin Elnour, Radwan Alsayed Radwan Ahmed, Ahmed Elkhalifa, Mohamed Eissa Elrayah Omran, Mohamed Ibrahim Osman Hamd, Rouida Elfadil Mohamed Ahmed Aboagla

**Affiliations:** 1 Neurosurgery, University Hospitals Coventry and Warwickshire National Health Service (NHS) Trust, Coventry, GBR; 2 General Surgery, Najran Armed Forces Hospital, Ministry of Defense Health Services, Najran, SAU; 3 General/Colorectal Surgery, Royal Oldham Hospital, Oldham, GBR; 4 Surgery, Dallah Hospital, Riyadh, SAU; 5 General Surgery, Almoweh General Hospital, Taif, SAU; 6 Pediatric Surgery, Ain Shams University Specialized Hospital, Cairo, EGY

**Keywords:** colorectal surgery, enhanced recovery after surgery, eras, length of stay, multimodal care, postoperative recovery

## Abstract

Colorectal surgery carries substantial risks of postoperative morbidity, extended hospital stays, and increased healthcare costs. While the overall benefits of enhanced recovery after surgery (ERAS) protocols are well established, recent studies have expanded their scope to include immunological outcomes, telemedicine integration, and patient-centered recovery metrics. This systematic review synthesizes the latest evidence from randomized controlled trials (RCTs) evaluating ERAS protocols in colorectal surgery, with particular attention to these emerging dimensions. A comprehensive search of PubMed, Scopus, Web of Science, Embase, and ClinicalTrials up to July 2025 identified 10 eligible RCTs from 414 screened records. Risk of bias was assessed using the Cochrane RoB 2 tool. Narrative synthesis was performed due to heterogeneity in ERAS components and outcome definitions. Consistent with prior literature, ERAS accelerated the return of bowel function, reduced the length of hospital stay, and lowered complication rates. Notably, recent trials demonstrated immunological benefits, including reductions in inflammatory markers (IL-6, CRP [C-reactive protein]) and preservation of immune function. Telemedicine-enhanced ERAS pathways, such as remote postoperative monitoring, further shortened recovery times while maintaining high patient satisfaction. Patient-centered outcomes, including functional independence, quality of life, and readiness for home discharge, were significantly improved. Most studies exhibited low risk of bias, although variability in ERAS implementation and reporting persisted. These findings confirm that contemporary ERAS protocols not only optimize physiological recovery but also address immune resilience, leverage digital health tools, and prioritize patient experience. Future research should standardize implementation and assess the long-term effects of these innovations.

## Introduction and background

Colorectal surgery is associated with significant physiological stress, postoperative complications, and prolonged hospital stays, all of which contribute to increased healthcare costs and patient morbidity [1]. Traditional perioperative care often involves prolonged fasting, aggressive fluid administration, and reliance on opioid analgesia, which may delay recovery and contribute to adverse outcomes [2]. In response to these challenges, the Enhanced Recovery After Surgery (ERAS) pathway was developed as a multimodal, evidence-based approach designed to attenuate surgical stress, accelerate recovery, and improve clinical outcomes [3].

ERAS protocols integrate preoperative, intraoperative, and postoperative interventions, including preoperative counseling, optimized nutrition, minimally invasive techniques, goal-directed fluid therapy, early enteral feeding, and prompt mobilization [4]. Since its inception, ERAS has been widely adopted in colorectal surgery, with numerous studies demonstrating reductions in postoperative ileus, length of hospital stay, and complication rates [5]. However, the degree of adherence to ERAS components, variations in protocol implementation, and heterogeneity in patient populations may influence its efficacy [6].

Despite the growing body of literature supporting ERAS, there remains a need for a comprehensive systematic review to evaluate its impact on postoperative recovery in colorectal surgery. Previous reviews have either focused on specific ERAS elements or included mixed surgical populations, potentially diluting the evidence specific to colorectal procedures. This systematic review aims to synthesize the latest evidence on the effectiveness of ERAS protocols in optimizing recovery outcomes, including postoperative complications, return of bowel function, hospital stay duration, and patient-reported outcomes. In addition, it examines emerging evidence on immunological benefits and the integration of telemedicine-based follow-up within ERAS pathways, providing a broader perspective on how these innovations enhance recovery and patient experience. By critically appraising the available data, this review will provide clinicians and policymakers with an updated assessment of ERAS efficacy and identify areas for future research to further refine perioperative care in colorectal surgery.

## Review

Methods

Study Design

This systematic review was conducted in accordance with the Preferred Reporting Items for Systematic Reviews and Meta-Analyses (PRISMA) 2020 guidelines.

Eligibility Criteria

This review included studies that met the following criteria: (1) randomized controlled trials (RCTs); (2) adult patients undergoing elective colorectal surgery; (3) intervention involving ERAS protocols compared with conventional perioperative care; and (4) reporting of at least one postoperative recovery outcome, such as length of hospital stay, postoperative complications, readmission rates, or return of bowel function. Studies were excluded if they were observational, non-randomized, case series, editorials, or reviews, or if ERAS components were not clearly defined or isolated.

Information Sources

A comprehensive search of five electronic databases was conducted: PubMed, Scopus, Web of Science, Embase, and ClinicalTrials. The last search was executed on July 03, 2025, with no restrictions on publication year or geographic location. Reference lists of included articles and relevant systematic reviews were manually screened to identify additional eligible studies.

Search Strategy

A tailored search strategy was developed for each database using a combination of Medical Subject Headings (MeSH) and relevant free-text keywords. Terms included “enhanced recovery,” “ERAS,” “colorectal surgery,” “colonic surgery,” “rectal surgery,” “randomized controlled trial,” and “postoperative recovery.” Boolean operators and truncations were used to improve sensitivity. The detailed strategy is present in the Appendix.

Selection Process

All identified records were imported into EndNote X9 (Clarivate Analytics, Philadelphia, PA, USA), a reference management software, and duplicates were removed. Two independent reviewers screened titles and abstracts against the inclusion criteria. Full texts of potentially eligible articles were retrieved and assessed for eligibility. Discrepancies during the selection process were resolved by discussion or consultation with a third reviewer.

Data Collection Process

Data were extracted independently by two reviewers using a pre-piloted standardized extraction form of Microsoft Excel (Microsoft Corporation, Redmond, WA, USA). Extracted data included study characteristics (authors, year, country, sample size), participant demographics, surgical approach (open or minimally invasive), ERAS components implemented, comparator details, primary and secondary outcomes, follow-up duration, and reported results. When necessary, study authors were contacted to clarify or provide missing data.

Data Items

The primary outcomes of interest were postoperative recovery metrics, including length of hospital stay, complication rates, readmission rates, time to return of bowel function, and mortality. Secondary outcomes included patient satisfaction, time to oral intake, and postoperative pain scores. Where multiple time points were reported, the most relevant or final outcome was extracted.

Study Risk of Bias Assessment

Risk of bias was assessed using the Revised Cochrane Risk-of-Bias Tool for Randomized Trials (ROB 2) [7]. This tool evaluates potential bias across five domains: the randomization process, deviations from intended interventions, missing outcome data, measurement of outcomes, and selection of the reported result. Two independent reviewers conducted the assessments. Discrepancies were resolved through consensus or by involving a third reviewer.

Effect Measures

Due to heterogeneity, no summary effect measures were calculated. Individual study findings were reported as presented by the original authors, including risk ratios, mean differences, and p-values where applicable.

Synthesis Methods

A quantitative meta-analysis was not conducted due to substantial heterogeneity across studies. This heterogeneity involved variability in ERAS protocol components, differences in surgical approach (open versus laparoscopic), inconsistent definitions and reporting of outcomes, and variation in follow-up periods. As a result, a narrative synthesis approach was employed, with findings organized thematically and supported by tabulated data to aid comparison and interpretation.

Reporting Bias Assessment

No formal statistical assessment of reporting bias was performed, as meta-analysis was not conducted. However, selective reporting was evaluated as part of the ROB 2 assessment.

Certainty Assessment

Assessment of the certainty of evidence (e.g., using GRADE) was not performed due to the narrative nature of synthesis and the lack of quantitative pooling.

Results

Study Selection Process

**Figure 1:**
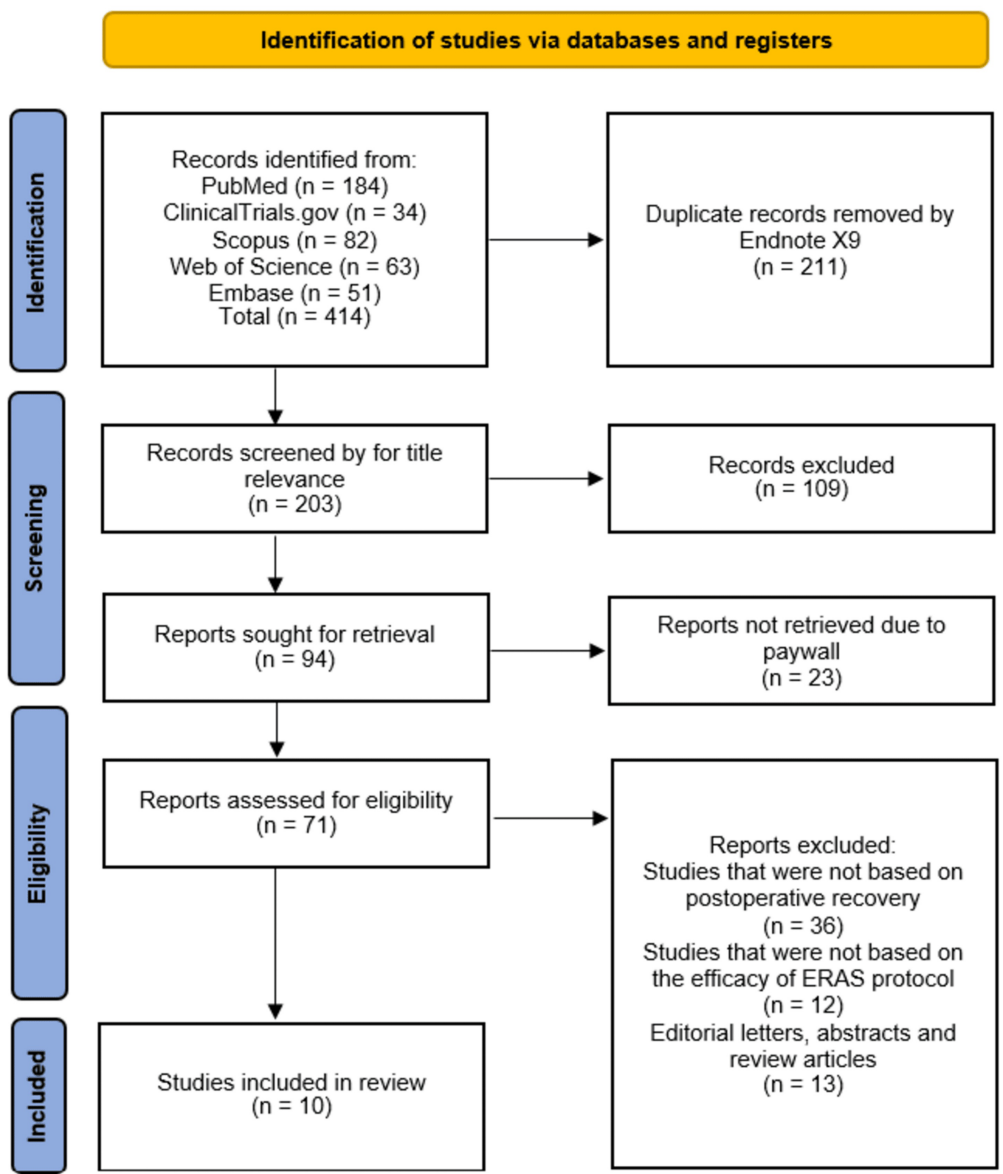
PRISMA 2020 flow diagram outlining literature search and inclusion steps PRISMA: Preferred Reporting Items for Systematic Reviews and Meta-Analyses

The study selection process followed the PRISMA guidelines and is summarized in the attached flowchart. A total of 414 records were initially identified from five databases: PubMed (n = 184), ClinicalTrials (n = 34), Scopus (n = 82), Web of Science (n = 63), and Embase (n = 51). After removing 211 duplicate records, 203 studies underwent title and abstract screening, of which 109 were excluded for irrelevance. The remaining 94 full-text articles were sought for retrieval; however, 23 were inaccessible due to paywall restrictions. We attempted to obtain these through author correspondence and institutional library requests, but were unsuccessful. Of the 71 assessed for eligibility, 36 were excluded for not focusing on postoperative recovery, 12 for not evaluating ERAS protocols, and 13 for being editorial letters, abstracts, or review articles. Ten studies [8-17] met the inclusion criteria and were included in this systematic review (Figure 1).

Study Characteristics

The systematic review included 10 studies [8-17] evaluating the efficacy of ERAS protocols in colorectal surgery, comprising RCT studies from diverse geographical regions, including Pakistan, Switzerland, China, Italy, Korea, Egypt, Norway, and the USA (Table 1). Sample sizes ranged from 30 to 200 participants, with both laparoscopic and open surgical approaches represented. Populations varied from general adult patients to elderly cohorts (≥70 years) and those with colorectal cancer. ERAS protocols were compared to conventional perioperative care, with primary outcomes focusing on postoperative recovery metrics such as time to bowel function recovery, length of stay (LOS), complication rates, and inflammatory markers.

**Table 1:** Key characteristics of studies included in this systematic review

Author (Year)	Country	Study Design	Sample Size (ERAS/Control)	Population Characteristics	Type of Surgery	ERAS Components Implemented	Primary Outcomes	Follow-Up Duration	Key Findings
Iqbal et al., [8] (2024)	Pakistan	RCT	60 (30 ERAS / 30 Control)	Patients undergoing elective colorectal surgery	Elective colorectal surgery	Perioperative strategies including early return of bowel function, reduced hospital stay, early flatus	Time to return of bowel sounds, time to first flatus, length of hospital stay, surgical site infection	4 weeks	ERAS group had significantly faster return of bowel function, shorter hospital stay, and fewer surgical site infections compared to conventional care
Ostermann et al., [9] (2019)	Switzerland	RCT (non-blinded)	75/75	Elderly patients (≥70 years) undergoing elective colorectal surgery	Elective colorectal surgery	Multimodal, multidisciplinary care: reduced perioperative stress, opioid-sparing pain control, early discharge, independence preservation, high compliance (77.2%)	30-day postoperative morbidity	30 days	ERAS reduced morbidity by 47%, fewer total and infectious complications, no anastomotic leaks, shorter hospital stay (7 vs 12 days), and better independence (87% vs 67% home discharge)
Feng et al., [10] (2016)	China	Prospective RCT	116 / 114	Patients undergoing colorectal surgery (Aug 2014 – Mar 2015)	Colorectal surgery	Early oral intake, early ambulation, reduced fasting, possibly minimal invasive handling (inferred as part of FTS)	Inflammatory mediators (CRP, IL-6, TNF-α), immunological indicators (IgG, IgA, C3, C4), recovery indices, complications	NR	FTS group showed lower inflammation, better immune response, faster functional recovery, and fewer complications compared to control
Mari et al., [11] (2016)	Italy	RCT	70 / 70	Patients undergoing elective major colorectal laparoscopic surgery	Elective major colorectal laparoscopic surgery	ERAS protocol	Immune and nutritional biomarkers (IL-6, CRP, cortisol, etc.), short-term outcomes	5 days	ERAS reduced IL-6 and CRP levels significantly postoperatively and improved prealbumin synthesis; no differences in cortisol and prolactin levels
Taupyk et al., [12] (2015)	Korea	Blinded RCT	31 / 39	Patients with colorectal cancer undergoing laparoscopic resection	Laparoscopic colorectal resection	Skipping pre-operative mechanical bowel prep, early restoration of diet, early post-operative ambulation	Length of hospital stay, bowel function recovery (flatus, defecation, solid diet), CRP levels, complications	NR	FTS significantly shortened hospital stay and improved bowel recovery, with lower CRP levels and no increase in complications.
Shetiwy et al., [13] (2017)	Egypt	RCT	70 (35/35)	Patients planned for elective laparoscopic colorectal resection for colorectal cancer	Elective laparoscopic colorectal resection	Early removal of nasogastric tube, early enteral feeding, early drain removal, evidence-based perioperative elements (unspecified), standardized care pathway	Length of hospital stay	NR	ERAS group had significantly shorter hospital stay, earlier NGT removal, earlier feeding and drain removal, fewer complications, with similar readmission rates
Abd ElRahman et al., [14] (2020)	Egypt	Prospective multicenter RCT	40/40	Adult patients with left-sided colonic cancer undergoing elective open resection	Elective open left-sided colonic cancer resection	Pain management, antiemetic use, early mobilization, shortened fasting	Hospital stay, postoperative pain, nausea/vomiting, complications, readmissions	≥1 month	ERP significantly reduced pain, nausea/vomiting, and hospital stay (P
Li et al., [15] (2019)	China	RCT	100 / 100	Patients undergoing laparoscopic radical surgery for colorectal cancer (June 2014–June 2017)	Laparoscopic radical resection of colorectal cancer	Perioperative ERAS components (e.g., early mobilization, early feeding, minimized tube use)	Operation time, blood loss, first exhaust/defecation time, extubation time, complication rate, VAS scores, nutritional status	7 days postoperatively (for outcome assessments)	ERAS significantly improved GI recovery, reduced complications, and improved nutritional markers compared to conventional care
Forsmo et al., [16] (2016)	Norway	Prospective single-center RCT	61 / 61	Adults undergoing laparoscopic or open colorectal resection with planned stoma	Colorectal resection with stoma formation	Pre- and postoperative stoma education, counselling, dedicated ERAS and stoma nurse specialists	Total postoperative hospital stay	30 days	ERAS group had significantly shorter hospital stay (6 vs. 9 days, p<0.001); no significant differences in morbidity, re-admissions, stoma complications, or 30-day mortality
Bednarski et al., [17] (2019)	USA	Phase II Prospective RCT	14 / 16	Adults aged 18–80 undergoing minimally invasive colorectal resection	Robotic or laparoscopic colectomy	Standard ERP + TeleRecovery (accelerated POD 1 discharge, televideoconference on POD 2)	Total 30-day length of stay (LOS)	30 days	TeleRecovery + ERP + MIS reduced 30-day LOS significantly (28.3h vs. 51.5h; P=0.041) with no increase in adverse events; patient satisfaction and QoL were preserved.

Key ERAS Components

The ERAS protocols across studies incorporated multimodal interventions tailored to perioperative care (Table 2). Common components included early mobilization [10,15], early oral intake [12,13], opioid-sparing pain management [9], and minimized use of invasive procedures (e.g., nasogastric tube removal, reduced mechanical bowel preparation) [12,13]. Specialized elements, such as stoma education [16] and TeleRecovery programs [17], were also highlighted. Compliance with ERAS protocols was explicitly reported in some studies, such as Ostermann et al. [9], where adherence reached 77.2%.

**Table 2:** ERAS components in included studies ERAS: Enhanced Recovery After Surgery

Author (Year)	Number of ERAS Components Used	Surgical Approach	Key ERAS Interventions
Iqbal et al., [8] (2024)	>1 component	Elective Colorectal Surgery	Early return of bowel function, reduced time to first flatus, reduced hospital stay, infection monitoring
Ostermann et al., [9] (2019)	compliance 77.2%	Elective colorectal surgery	Multimodal care approach including: reduction of perioperative stress, pain management (reduced opioid use), infection prevention, early discharge, and independence preservation
Feng et al., [10] (2016)	≥4 inferred	NR	Early oral intake, early ambulation, early defecation, early aerofluxus (bowel movement), reduced inflammation and immune optimization (suggesting multimodal perioperative care)
Mari et al., [11] (2016)	Multimodal	Laparoscopic colorectal surgery	Nutritional optimization (e.g., improved prealbumin levels), modulation of inflammatory response (e.g., reduced IL-6 and CRP), early recovery pathway elements implied by ERAS
Taupyk et al., [12] (2015)	3	Laparoscopic Colorectal Resection	- No pre-operative mechanical bowel preparation- Early restoration of diet- Early post-operative ambulation
Shetiwy et al., [13] (2017)	At least 4 identifiable components	Elective laparoscopic colorectal resection	Early removal of nasogastric tubes, early enteral feeding, early removal of intra-abdominal drains, standardized perioperative care pathway components
Abd ElRahman et al., [14] (2020)	Implied multiple	Open left-sided colonic surgery	- Pain management- Reduction of postoperative nausea and vomiting (PONV)- Shortened hospital stay
Li et al., [15] (2019)	Multiple components inferred	Laparoscopic radical resection of colorectal cancer	Early extubation, early ambulation, early feeding (inferred from shortened first exhaust and defecation time), nutritional support, complication prevention
Forsmo et al., [16] (2016)	≥2 inferred	Laparoscopic or open surgery	Pre-operative and postoperative stoma education; counselling by dedicated ERAS/stoma nurses
Bednarski et al., [17] (2019)	Include standard ERP components + accelerated discharge + TeleRecovery	Robotic (21 patients), Laparoscopic (9 patients)	- Minimally invasive colorectal resection- Enhanced Recovery Protocol (ERP)- Accelerated discharge on POD 1- TeleRecovery (video consultation on POD 2)

Postoperative Outcomes

Recovery of bowel function: ERAS protocols significantly accelerated the return of bowel function. Iqbal et al. [8] reported faster recovery of bowel sounds and first flatus in the ERAS group compared to conventional care (p < 0.05). Similarly, Li et al. [15] observed reduced time to first exhaust and defecation (p < 0.01), while Taupyk et al. [12] noted improved bowel recovery without increased complications.

Length of hospital stay: ERAS consistently reduced LOS across studies. Ostermann et al. [9] demonstrated a five-day reduction in LOS (7 vs. 12 days, p < 0.001) for elderly patients, while Forsmo et al. [16] reported a three-day reduction (6 vs. 9 days, p < 0.001). Bednarski et al. [17] achieved the shortest LOS (28.3 hours vs. 51.5 hours, p = 0.041) using an accelerated discharge protocol combined with telemedicine.

Complications and inflammatory response: ERAS protocols were associated with lower morbidity and improved immune responses. Ostermann et al. [9] reported a 47% reduction in 30-day morbidity, with fewer infectious complications. Mari et al. [11] and Feng et al. [10] documented reduced inflammatory markers (IL-6, CRP) and better immune profiles (e.g., higher IgG, IgA) in ERAS groups. Abd ElRahman et al. [14] noted significant reductions in postoperative pain and nausea/vomiting (p < 0.001) without increased readmissions.

Functional recovery and patient-centered outcomes: ERAS improved functional independence, particularly in elderly patients (87% vs. 67% home discharge, Ostermann et al. [9]). Bednarski et al. [17] preserved patient satisfaction and quality of life despite accelerated discharge, while Forsmo et al. [16] emphasized the role of stoma education in enhancing recovery.

Heterogeneity and Limitations

Variability in ERAS components (e.g., number of interventions, compliance rates) and surgical techniques (laparoscopic vs. open) was observed. Some studies lacked detailed descriptions of control group protocols [10], and follow-up durations varied (4 weeks to 30 days). Nonetheless, the collective findings underscore ERAS as a robust framework for optimizing postoperative recovery in colorectal surgery.

Results of Risk of Bias Assessment

The risk of bias assessment, conducted using the Cochrane RoB 2 tool, revealed that the majority of included studies demonstrated low risk of bias across all domains, including randomization, deviations from intended interventions, missing outcome data, measurement of outcomes, and selective reporting. Specifically, Iqbal et al. [8], Ostermann et al. [9], Mari et al. [11], Taupyk et al. [12], Abd ElRahman et al. [14], Li et al. [15], Forsmo et al. [16], and Bednarski et al. [17] were judged as low risk overall, indicating robust methodological quality. However, Feng et al. [10] and Shetiwy et al. [13] raised some concerns, primarily due to unclear allocation concealment in the randomization process [10] and potential bias in outcome measurement [13]. No studies were rated as high risk, underscoring the reliability of the synthesized evidence in this systematic review (Table 3). The consistency in low bias risk across most studies strengthens confidence in the validity of the findings regarding ERAS protocols in colorectal surgery.

**Table 3:** Risk of bias assessment using Cochrane ROB 2 tool ROB 2: Revised Cochrane Risk-of-Bias Tool for Randomized Trials

Study (Author, Year)	Randomization Process	Deviations from Intended Interventions	Missing Outcome Data	Measurement of the Outcome	Selection of Reported Result	Overall Risk of Bias
Iqbal et al., [8] (2024)	Low risk	Low risk	Low risk	Low risk	Low risk	Low risk
Ostermann et al., [9] (2019)	Low risk	Low risk	Low risk	Low risk	Low risk	Low risk
Feng et al., [10] (2016)	Some concerns	Low risk	Low risk	Some concerns	Low risk	Some concerns
Mari et al., [11] (2016)	Low risk	Low risk	Low risk	Low risk	Low risk	Low risk
Taupyk et al., [12] (2015)	Low risk	Low risk	Low risk	Low risk	Low risk	Low risk
Shetiwy et al., [13] (2017)	Some concerns	Some concerns	Low risk	Some concerns	Low risk	Some concerns
Abd ElRahman et al., [14] (2020)	Low risk	Low risk	Low risk	Low risk	Low risk	Low risk
Li et al., [15] (2019)	Low risk	Low risk	Low risk	Low risk	Low risk	Low risk
Forsmo et al., [16] (2016)	Low risk	Low risk	Low risk	Low risk	Low risk	Low risk
Bednarski et al., [17] (2019)	Low risk	Low risk	Low risk	Low risk	Low risk	Low risk

Discussion

The findings of this systematic review underscore the significant benefits of ERAS protocols in optimizing postoperative recovery for patients undergoing colorectal surgery. Across the 10 included studies, ERAS demonstrated consistent improvements in key recovery metrics, including accelerated return of bowel function, reduced length of LOS, lower complication rates, and enhanced patient-centered outcomes. These results align with the broader literature on ERAS, which has increasingly been recognized as a transformative approach in perioperative care [18]. For instance, the faster recovery of bowel function observed in studies such as Iqbal et al. [8] and Li et al. [15] corroborates earlier findings by Gustafsson et al. [19], who reported that early mobilization and oral intake, core ERAS components, stimulate gastrointestinal motility and reduce postoperative ileus. Similarly, the reduction in LOS, as demonstrated by Ostermann et al. [9] and Bednarski et al. [17], mirrors meta-analytic evidence showing that ERAS protocols can shorten hospitalization by 2-3 days without increasing readmission rates [20]. This consistency across diverse populations, including elderly patients [9] and those undergoing minimally invasive surgery [17], highlights the adaptability and scalability of ERAS principles.

A particularly compelling aspect of the reviewed studies is the multimodal nature of ERAS interventions, which collectively address physiological, immunological, and psychological stressors associated with surgery. For example, the reduction in inflammatory markers (IL-6, CRP) reported by Mari et al. [11] and Feng et al. [10] suggests that ERAS mitigates the surgical stress response, a phenomenon well-documented in prior research. The integration of opioid-sparing pain management, as seen in Ostermann et al. [9], further aligns with evidence that minimizing opioid use reduces postoperative nausea, vomiting, and ileus [21]. Moreover, specialized interventions like stoma education [16] and TeleRecovery programs [17] exemplify how ERAS can be tailored to address specific patient needs, reinforcing its patient-centered ethos. These findings resonate with the broader shift toward value-based care, where ERAS has been shown to improve clinical outcomes while reducing healthcare costs [22].

However, the review also reveals heterogeneity in ERAS implementation, which warrants careful interpretation. While most studies reported high compliance rates (e.g., 77.2% in Ostermann et al. [9]), others, such as Feng et al. [10], provided limited details on protocol adherence. This variability echoes challenges identified in large ERAS registries, where inconsistent application of components can dilute outcomes [23]. For instance, the absence of standardized control group protocols in some studies [10] complicates direct comparisons, a limitation noted in previous meta-analyses [24]. Additionally, the predominance of laparoscopic approaches in studies like Taupyk et al. [12] and Li et al. [15] raises questions about the generalizability of findings to open surgeries, though Abd ElRahman et al. [14] demonstrated ERAS efficacy in open resections. These nuances highlight the need for standardized reporting and protocol fidelity in future research.

The immunological benefits of ERAS, particularly the modulation of inflammatory responses, further validate its physiological rationale. The marked reduction in IL-6 and CRP levels observed by Mari et al. [11] and Feng et al. [10] supports the hypothesis that ERAS attenuates surgical stress, thereby preserving immune function. This is critical in colorectal surgery, where postoperative immunosuppression increases infection risks. The improved nutritional outcomes, such as higher prealbumin levels in Mari et al. [11], also align with evidence that early enteral feeding enhances recovery [25]. These mechanistic insights strengthen the case for ERAS as not merely a logistical pathway but a biologically grounded strategy to optimize recovery.

Patient-centered outcomes, such as functional independence and quality of life, emerged as another strength of ERAS. The high rate of home discharge among elderly patients in Ostermann et al. [9] (87% vs. 67%) reflects ERAS’s potential to preserve autonomy, a finding consistent with studies by McLeod et al. [26]. Similarly, Bednarski et al. [17] demonstrated that telemedicine-integrated ERAS maintained patient satisfaction despite accelerated discharge, addressing concerns about the trade-off between early discharge and care quality. These results challenge traditional postoperative paradigms, suggesting that ERAS can achieve both efficiency and patient satisfaction, a dual imperative in modern healthcare [27].

Limitations****

Limitations of the Evidence Base

Despite the robust evidence, several limitations must be acknowledged. First, the variability in ERAS components and compliance across studies complicates pooled interpretations. Second, the predominance of RCTs from high-income settings may limit generalizability to resource-limited contexts. Third, short follow-up durations in studies like Li et al. [15] (seven days) preclude assessment of long-term outcomes, such as cancer recurrence or chronic pain. Finally, the lack of blinding in most trials, while inherent to ERAS studies, introduces performance bias.

Limitations of This Review

This review also has certain methodological limitations. A potential selection bias may exist due to the inability to access 23 potentially eligible articles despite attempts through author correspondence and institutional library requests. Two included studies had “some concerns” in the risk of bias assessment, which may have influenced specific outcome interpretations. The narrative synthesis approach, necessitated by substantial heterogeneity in ERAS protocols and outcome measures, limited the ability to perform quantitative pooling or certainty grading. Additionally, the possibility of publication bias cannot be excluded, as unpublished or negative studies may not have been captured.

## Conclusions

ERAS protocols significantly enhance postoperative recovery in colorectal surgery. By integrating multimodal interventions, ERAS mitigates physiological stress, accelerates functional recovery, and improves patient-centered outcomes. While heterogeneity in implementation and reporting underscores the need for standardization, the consistency of benefits across diverse populations and settings affirms ERAS as a best-practice framework. Future research should prioritize long-term outcomes, cost-effectiveness analyses, and strategies to optimize protocol adherence in real-world practice.

## Appendices

**Table 4:** Detailed search strategy for each database

Database	Search strategy
PubMed (MEDLINE via PubMed)	Concept A = ERAS: ("Enhanced Recovery After Surgery"[Mesh] OR "Enhanced Recovery After Surgery"[tiab] OR ERAS[tiab] OR "fast track"[tiab] OR "fast-track"[tiab] OR "fast track surgery"[tiab])Concept B = colorectal surgery: ("Colorectal Surgery"[Mesh] OR "Colorectal Neoplasms"[Mesh] OR colorectal[tiab] OR colonic[tiab] OR colon[tiab] OR rectal[tiab] OR rectum[tiab] OR "colorectal surgery"[tiab] OR "colon surgery"[tiab] OR "rectal surgery"[tiab])Combined: (#A) AND (#B)
Embase (Ovid or Embase.com)	(EMTREE + keywords)('enhanced recovery after surgery'/exp OR 'enhanced recovery after surgery':ti,ab,kw OR ERAS:ti,ab,kw OR 'fast track':ti,ab,kw OR 'fast-track':ti,ab,kw)AND('colorectal surgery'/exp OR 'colorectal':ti,ab,kw OR colon*:ti,ab,kw OR colonic:ti,ab,kw OR rectal:ti,ab,kw OR 'rectum':ti,ab,kw OR 'colorectal surgery':ti,ab,kw)
Scopus	TITLE-ABS-KEY( "enhanced recovery after surgery" OR ERAS OR "fast track" OR "fast-track" OR "fast track surgery" )ANDTITLE-ABS-KEY( colorectal OR colon OR colonic OR rectal OR rectum OR "colorectal surgery" OR "colon surgery" OR "rectal surgery")
Web of Science (Core Collection)	TS=("enhanced recovery after surgery" OR ERAS OR "fast track" OR "fast-track")ANDTS=(colorectal OR colon OR colonic OR rectal OR rectum OR "colorectal surgery" OR "colon surgery" OR "rectal surgery")
ClinicalTrials.gov	Basic advanced query (enter in the search box or Advanced Search fields):(Enhanced Recovery OR "Enhanced Recovery After Surgery" OR ERAS OR "fast track" OR "fast-track") AND (colorectal OR colon OR rectal OR "colorectal surgery")Or use the Advanced Search: Interventions/Keywords = ERAS OR enhanced recovery; Condition/Disease = colorectal OR colon OR rectal
